# Development of a high-performance thin-layer chromatography-based method for targeted glycerolipidome profiling of microalgae

**DOI:** 10.1007/s00216-023-05101-y

**Published:** 2024-01-04

**Authors:** Kolos Makay, Carola Griehl, Claudia Grewe

**Affiliations:** 1 Research Group of Bioprocess Engineering, Center of Life Sciences of Anhalt University of Applied Sciences, Bernburger Str. 55, 06366 Köthen, Germany; 2 Competence Center Algal Biotechnology, Anhalt University of Applied Sciences, Bernburger Str. 55, 06366 Köthen, Germany

**Keywords:** Microalgae, Quantitative HPTLC, Glycerolipids, PUFAs

## Abstract

The conditionally essential very-long-chain polyunsaturated fatty acids (VLC-PUFAs), such as eicosapentaenoic acid (EPA, C20:5 n-3), play a vital role in human nutrition. Their biological activity is thereby greatly influenced by the distinct glycerolipid molecule that they are esterified to. Here, microalgae differ from the conventional source, fish oil, both in quantity and distribution of VLC-PUFAs among the glycerolipidome. Therefore, the aim of this study was to develop a fast and reliable one-dimensional high-performance thin-layer chromatography (HPTLC)-based method that allows the separation and quantification of the main microalgal glycerolipid classes (e.g., monogalactosyldiacylglycerol (MGDG), sulfoquinovosyl diacylglycerol (SQDG), phosphatidylglycerol (PG)), as well as the subsequent analysis of their respective fatty acid distribution via gas chromatography (GC) coupled to mass spectrometry (MS). Following optimization, method validation was carried out for 13 different lipid classes, based on the International Conference on Harmonization (ICH) guidelines. In HPTLC, linearity was effective between 100 and 2100 ng, with a limit of quantification between 62.99 and 90.09 ng depending on the glycerolipid class, with strong correlation coefficients (*R*^2^ > 0.995). The recovery varied between 93.17 and 108.12%, while the inter-day precision measurements showed coefficients of variation of less than 8.85%, close to the limit of detection. Applying this method to crude lipid extracts of four EPA producing microalgae of commercial interest, the content of different glycerolipid classes was assessed together with the respective FA distribution subsequent to band elution. The results showed that the described precise and accurate HPTLC method offers the possibility to be used routinely to follow variations in the glycerolipid class levels throughout strain screening, cultivation, or bioprocessing. Thus, additional quantitative analytical information on the complex lipidome of microalgae can be obtained, especially for n-3 and n-6 enriched lipid fractions.

**Graphical Abstract:**

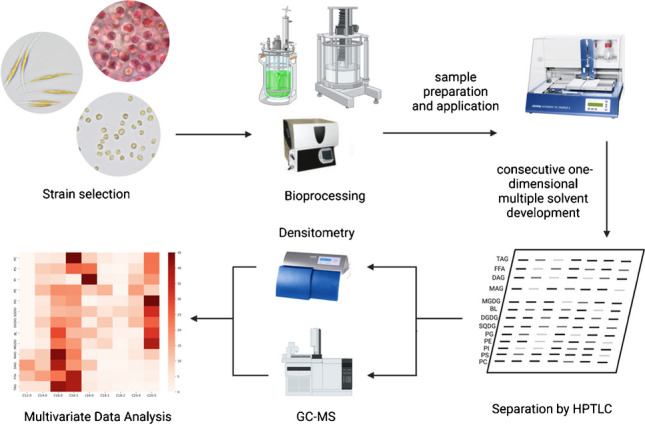

**Supplementary Information:**

The online version contains supplementary material available at 10.1007/s00216-023-05101-y.

## Introduction

Microalgae are a diverse group of primarily unicellular and phototrophic organisms. Owing to this one-cell-concentrated complex metabolism, microalgae are considered suitable candidates for producing multiple products (e.g., carotenoids, polysaccharides, fatty acids, and polyphenols) with diverse biological activities (e.g., antioxidant, anti-inflammatory, antitumor, and antimicrobial activities) [1, 2]. Among these cellular products, lead compounds are of high interest, as they have shown sufficient potential (measured by potency, therapeutic efficacy, etc.) to progress into a full product development program in the field of life sciences (e.g., cosmeceuticals, nutraceuticals, and pharmaceutical industries) [3–7].

A well-known example of such lead compounds is the VLC-PUFAs, such as arachidonic acid (ARA, C20:4 n-6), EPA, and docosahexaenoic acid (DHA, C22:6 n-3) [8, 9]. In addition to being building blocks of cellular membranes, PUFAs have been shown to play a significant role as mediator molecules [10] in pre- and postnatal brain development [11], regulation of cytokine storms [12], antidepressant mechanisms [13], and the prevention of cardiovascular diseases [14]. The de novo synthesis of n-3 and n-6 VLC-PUFAs is restricted to microorganisms, among which marine microalgae are considered rich and sustainable sources [15].

**Fig. 1:**
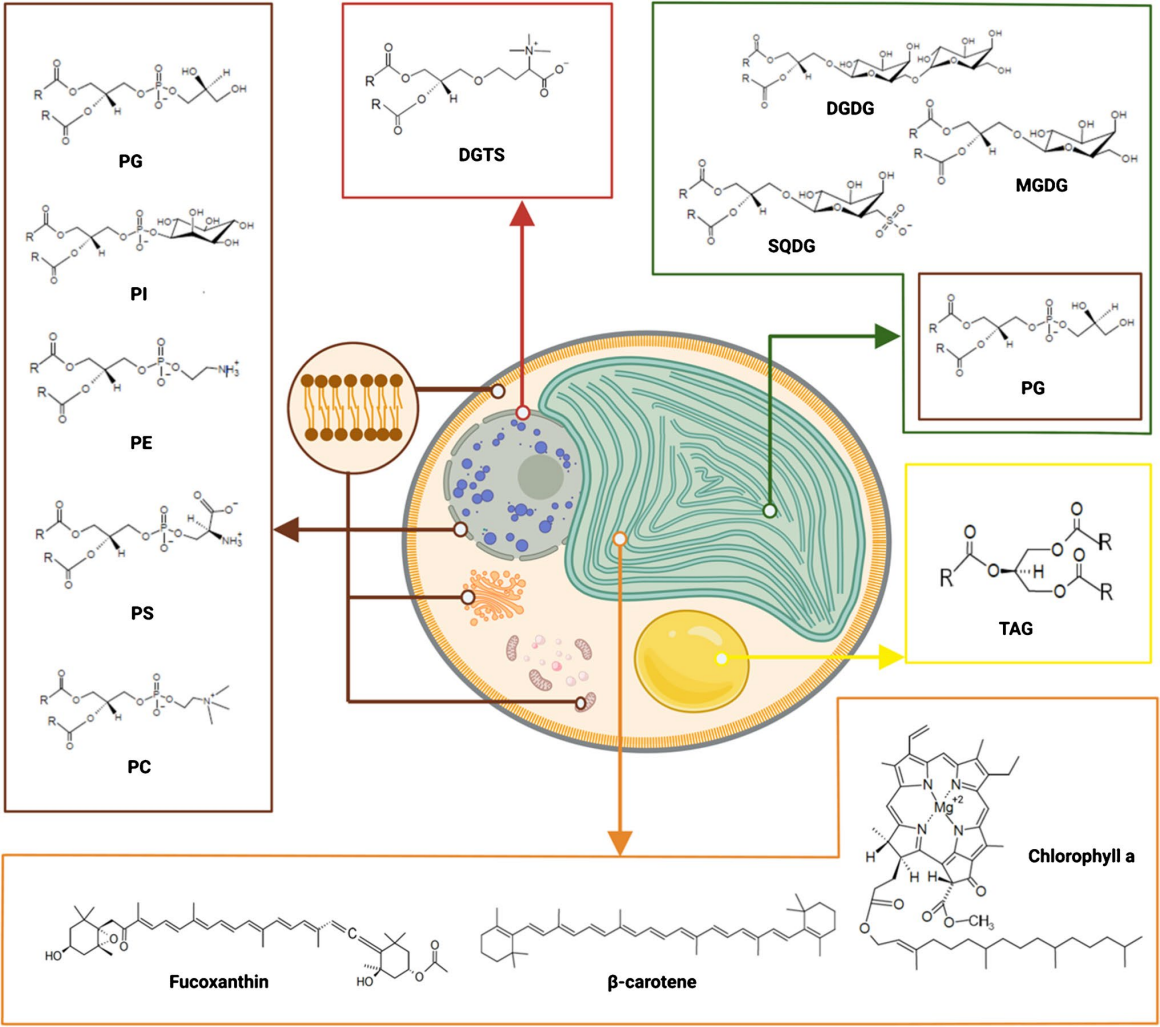
Schematic figure of a microalgae cell from the class Eustigmatophyceae (e.g., *Nannochloropsis* sp.), representing the localization of the major lipid constituents of the microalgal lipidome such as glycolipids (GL, green), phospholipids (PL, brown), special ether bond-based betaine lipids (BL, red), neutral lipid (NL, yellow), and the chlorophyll/carotenoid fraction (orange) (R represents fatty acid acyl chains with different length (12–22 carbon atoms, e.g., EPA (C20:5, n-3)). Monogalactosyldiacylglycerol (MGDG), digalactosyldiacylglycerol (DGDG), sulfoquinovosyl diacylglycerol (SQDG), phosphatidylglycerol (PG), phosphatidylcholines (PC), phosphatidylethanolamine (PE), phosphatidylinositol (PI), phosphatidylserine (PS), diacylglyceryl-trimethylhomoserine (DGTS), triacylglycerol (TAG). Created with BioRender.com

Principally, FAs are present in the glycerolipidome of cells, which refers to the complete set of glycerolipids in which two fatty acid acyl chains (R_1_ and R_2_) are esterified at the sn-1 and sn-2 positions on the glycerol backbone, whereas the third hydroxyl group of the glycerol molecule is esterified with headgroups of different polarities or a third fatty acid acyl chain (R_3_) (Fig. 1) [16, 17]. Microalgae synthesize several types of glycerolipids, which based on the chemical structure of the headgroup, can be subclassified into glycolipids (GL), phospholipids (PL), special ether bond-based betaine lipids (BL), and the neutral lipid (NL) fraction (Fig. 1).

The most common types of GL classes found in microalgae are two non-ionic (monogalactosyldiacylglycerol (MGDG) and digalactosyldiacylglycerol (DGDG)) and one ionic sulfonated constituent (sulfoquinovosyl diacylglycerol (SQDG)). These GLs, along with phosphatidylglycerol (PG), are the main constituents of chloroplast membranes in which the photosynthesis-related molecules such as chlorophylls (e.g., chlorophyll a) and carotenoids (e.g., fucoxanthin, β-carotene) are embedded. In addition to PG, phosphatidylcholines (PC), phosphatidylethanolamine (PE), phosphatidylinositol (PI), phosphatidylserine (PS), and ether bond-based BLs (diacylglyceryl-trimethylhomoserine (DGTS), diacylglyceryl-trimethyl-β-alanine (DGTA), and diacylglyceryl-carboxyhydroxymethylcholine (DGCC)) are the major components of the non-photosynthetic membranes of microalgal cells. Furthermore, in microalgae, the neutral lipid fraction contains storage products such as triacylglycerol (TAG), which form lipid droplets that serve as cellular carbon reservoirs or intermediates of lipid metabolism (e.g., monoacylglycerol (MAG), diacylglycerol (DAG), and free fatty acids (FFA)) [16, 17].

Among these lipid classes, n-3 and n-6 VLC-PUFAs in microalgae are predominantly reported to be esterified into the medium-polar GLs [18, 19]. These n-3 and n-6 VLC-PUFAs containing GL fractions have shown increased bioavailability and bioactivity (e.g., antitumor, antiproliferative, and anti-inflammatory activities) compared to the fish oil-derived triacylglycerol (TAG)-esterified form [20, 21]; however, this phenomenon is still not fully understood. Therefore, to fully exploit the aforementioned health-promoting activities of n-3 and n-6 VLC-PUFAs, it is crucial to gain a deeper understanding of glycerolipid composition and remodeling in microalgae.

The headgroup of the molecule not only affects the biological activity but also the physicochemical properties, such as polarity. The polarity of the headgroups varies across a broad range between the glycerolipid classes, making their analysis challenging. Previous studies have utilized conventional thin-layer chromatography (TLC) techniques, which have been shown to be promising owing to the flexibility of planar chromatographic methods for the qualitative and indirect quantitative analyses of glycerolipids from microalgae [22–25]; however, they suffer from several limitations. For instance, traditional TLC plates have large particle sizes (5 to 20 µm), which result in poor separation efficiency. Thus, two-dimensional developments have to be applied for the efficient separation of polar glycerolipid classes. Furthermore, fast densitometric quantification, despite the development of direct on-plate derivatization methods (e.g., primulin and modified copper sulfate reagent), remains challenging owing to the multidimensional Rf values. Therefore, the individual spots must be scratched off, followed by time-consuming and laborious derivatization processes for indirect quantification (e.g., fluorometric/colorimetric assays and GC-coupled flame ionization detection (FID) or MS-based measurements) [24, 26–28].

To date, ultra-high-performance liquid chromatography (UHPLC) with evaporative light-scattering detector (ELSD), charged aerosol detector (CAD), and MS-based methods is the most commonly applied methods in glycerolipidomic studies [29–33]. However, these systems can suffer from limited separation of complex lipid mixtures resulting from the co-elution of different lipid species [34], which is particularly problematic when analyzing chemically less-defined crude lipid extracts. Therefore, prior to analysis, either laborious or sample amount intensive pre-chromatographic solid-phase extraction is required, or multiple separations with different stationary phases (reverse and normal phases) are necessary to map the main glycerolipid classes of interest. Nevertheless, the so-called memory effect resulting from the irreversible absorption and the usage of volatile buffers have recently been shown to unintentionally influence the accuracy of quantitative determination via ELSD [35, 36].

Among the above-mentioned techniques, only LC–MS allows the determination of the FA composition of a single lipid molecule. Thus, LC–MS methods have shown significant advancements and became a preferred choice of analysis in the field of lipidomics. However, a notable difficulty arises from inherent differences in ionization yield and molar response of the MS analyzer across various lipid species: the necessity of utilization of internal standards. Due to the vast number of individual glycerolipid molecule species, to have a corresponding ^13^C-labeled internal standard for each one is not feasible. Such variance may pose a challenge for absolute mass-based quantification of the glycerolipids and also potentially introducing bias into data obtained through LC–MS analysis [33]. For instance, as it was shown by Jouhet et al. (2017) that the application of one MGDG species (18:0/18:0) as internal standard can result in up to threefold overestimation of this glycerolipid class [24].

However, TLC performance has improved over the years, leading to high-performance thin-layer chromatography (HPTLC). Automated sample application and decreased and more homogeneous silica gel particle size distribution (from 4 to 8 µm) improved the analytical efficiency (e.g., lower detection limits, increased separation efficiency), making it a powerful tool for the one-dimensional separation of glycerolipids from complex mixtures, such as crude lipid extracts [27, 28]. This one-dimensional separation overcomes the limitations of previously applied TLC methods for studying the glycerolipid classes and allows quantitative on-plate analysis of multiple samples simultaneously, which is not possible with other chromatographic techniques, including multiplex UHPLC systems [27, 28, 36–40].

In microalgal lipidomic research, separation of the major polar glycerolipids in a multidimensional manner via TLC and one-dimensional separation of the neutral lipid fractions are routinely performed using indirect quantification [22, 41]. However, the efficacy of separating and quantifying major glycerolipid lipid classes (Fig. 1) on one plate using a one-dimensional HPTLC-based scanning densitometry procedure has not yet been evaluated [28]. Therefore, the aim of this study was to develop and validate an HPTLC-based analytical method to enable precise and accurate analysis for a pre-defined set of 13 lipid classes. Subsequently, crude lipid extract of microalgae species across different classes (*Nannochloropsis granulata* (Eustigmatophyceae), *Phaeodactylum tricornutum* (Bacillariophyceae), *Porphyridium purpureum* (Rhodophyceae), and *Tetraselmis tetrathele* (Chlorodendrophyceae)) was subjected to this targeted glycerolipid class quantification and lipid class-specific fatty acid distribution. Although these species are of commercial interest for the production of n-3 and n-6 VLC-PUFAS [42] and their FA profiles have already been well explored, much less is known about the proportion and composition of single glycerolipid classes.

## Materials and methods

### Microalgae strains and cultivation

*Nannochloropsis granulata*, *Phaeodactylum tricornutum*, *Porphyridium purpureum*, and *Tetraselmis tetrathele* were obtained from the Competence Centre Algal Biotechnology (CAB, Köthen, Germany). The strains were maintained in cell culture flasks under natural light–dark cycles at room temperature (~ 22 ± 2 °C). *N. granulata* and *T. tetrathele* were maintained and cultivated in ½ ES1 medium [43] whereas *P. tricornutum* and *P. purpureum* in Mann and Myers [44] and P.C*.* [45] medium, respectively*.* All species were cultivated in 4-L bubble columns under continuous illumination (*I*_0_ = 186 μmol m^−2^ s^−1^ light measured at the inner surface of the bioreactor). The culture was tempered at 22 °C and aerated at 0.45 vvm with 2% (v/v) CO_2_. Cultivation was performed as a semi-continuous batch. The starting biomass concentration was 0.32 ± 0.05 g L^−1^, and the microalgae were cultivated for 7 days. The biomass was harvested and centrifuged at 4.225 × g for 10 min at 4 °C. The obtained pellets were washed twice with deionized water and freeze-dried. The dried algal powder was transferred into glass vials under a nitrogen stream, sealed, and stored at − 85 °C until glycerolipidome analysis.

### Determination of the residual moisture content

The residual moisture content of the lyophilized algal biomass was determined gravimetrically. Lyophilized biomass (20 mg) was transferred to a pre-weighed glass tube and dried at 105 °C for 24 h. After the tubes were cooled to room temperature in a desiccator, the residual moisture content of the biomass was calculated.

### Determination of total lipid content

One hundred milligrams of lyophilized algal biomass was mixed with approximately 0.5-mL (diameter, 1.0 mm) and 0.3-mL (diameter, 0.1 mm) glass beads to which 100 μL of chloroform:methanol (2:1, v/v) was pipetted and vortexed thoroughly. Biomass was disrupted for 10 min at a frequency of 30 s^−1^ by the vibration mill MM400 (Retsch, Haan, Germany). For the disintegrated biomass, 1 mL of ice-chilled chloroform:methanol (2:1, v/v) was pipetted, vortexed thoroughly, and centrifuged for 1 min at 15,100 g. The supernatant was transferred into a 10-mL glass tube. The extraction process was repeated nine times and performed on ice in the absence of light. To the pooled lipid extract, 2 mL of 1 M NaCl solution was added and mixed thoroughly. After phase separation, the lower organic phase was collected in a pre-weighed glass tube, evaporated using a Hei-VAP Core HL G1 rotary evaporator (Heidolph, Schwabach, Germany), and dried at 105 °C for 24 h. After drying, the tubes were cooled to room temperature in a desiccator, the weight of the glass tube was measured, and the lipid content of the dry algal powder was calculated.

### HPTLC

#### Standards and chemicals

Analytical grade organic solvents used in this study along with HPTLC chromatography plates (20 × 10 and 10 × 10 cm silica gel 60 F_254_ glass plates) were obtained from Merck (Darmstadt, Germany). Copper sulfate heptahydrate, potassium chloride, orthophosphoric acid (85%, w/w), and sulfuric acid (96% w/w) were obtained from CarlRoth (Karlsruhe, Germany). Lipid standards, namely, phosphatidylcholine (PC 15:0–18:1), phosphatidylethanolamine (PE 15:0–18:1), phosphatidylinositol (PI 18:1–18:1), phosphatidylserine (PS 18:1–18:1), phosphatidylglycerol (PG 16:0–18:1), diacylglyceryl trimethyl-homoserin (DGTS 16:0–16:0), monogalactosyldiacylglycerol (MGDG 18:1–18:1), digalactosyldiacylglycerol (DGDG 18:3–18:3 as main species), sulfoquinovosyldiacylglycerol (SQDG 18:3–16:0 as main species), eicosapentaenoic acid as a free fatty acid (FA), triolein as a triacylglycerol (TAG), di-oleic acid as a diacylglycerol (DAG), and monooleic acid as a monoacylglycerol (MAG), were purchased from Merck. The lipid standards were dissolved in n-hexane (p.a. ≥ 99%) and sonicated for 30 s. Stock solutions were prepared at a concentration of 1 mg mL^−1^ and aliquoted into 1.5-mL vials under a nitrogen stream, sealed, and stored at − 85 °C until analysis.

## Method development

### Pre-washing and plate activation

HPTLC silica gel 60 F_254_ plates were pre-washed in a glass development chamber with isopropanol, until the frontline reached 19 or 9 cm, deepening on the plate used. After drying, the plates were activated in an oven at 130 °C for 60 min.

### Preparation of microalgae samples for HPTLC analysis

Lyophilized algae samples for HPTLC analysis were prepared as described in the “Determination of total lipid content” section. However, 10 mg of lyophilized algae powder was used, and following the washing step and phase separation with 1 M NaCl, the lower organic phase was collected and filtered through a PTFE membrane (0.22 µm) into a volumetric flask. The final volume of the lipid extract was adjusted to 5 mL using chloroform:methanol (2:1, v/v) and was analyzed directly.

### Sample application

Samples and standards were applied using Linomat 5 (CAMAG, Muttenz, Switzerland), controlled by the software vivsionCATS (CAMAG, Muttenz, Switzerland), at 10 mm of distance from the bottom of the plate, as 8-mm-long bands, with 11.4 mm center to center distance from each other. A constant application rate of 150 nL s^−1^ was used under continuous drying with a stream of compressed at 6 bars. Lipid standards, stock solutions (1 mg mL^−1^) resulting in an amount of 100 …., 3100 ng band^−1^, were loaded onto the plates with an equidistant increase in concentration. The samples were applied at different volumes (2 to 10 µL) because of the different contents of the targeted lipid compounds in the cells.

### Plate development

Based on the evaluation of the available literature and own experiments (see “Method development”), the mobile phase systems of methyl acetate:isopropanol:chloroform:methanol:0.25% KCl were chosen, and the ratio of the applied solvents was optimized for the separation of the more polar PL and GL lipid classes. The final composition of the first mobile phase was methyl acetate:isopropanol:chloroform:methanol:0.25% aqueous KCl solution (w/w%, acidified with glacial acetic acid) in the ratio of 25:25:25:10:4,35 (v/v/v/v), and the plate was developed up to a height of 90 mm. The plate was dried in a stream of cold air, and a second mobile phase system, consisting of n-hexane:acetone:isopropanol at a ratio of 80:20:5 (v/v/v), was applied up to 120 mm from the bottom of the plate. As the third mobile phase system, n-hexane:diethyl:ether:glacial acetic acid (70:30:1, v/v/v) was applied and developed until the frontline reached 170 mm. Before development, the glass chambers were saturated with freshly prepared mobile phase for at least 60 min at 22 ± 2 °C. For the saturation of the chambers, 20 mL of mobile phase system was used in each trial, so the HPTLC plates were wetted to 5 mm each time at the start of the chromatographic development.

### Derivatization and detection

For quantitative detection, the developed plates were dipped for 6 s in a modified copper sulfate reagent [46], which was prepared by dissolving 20 g of CuSO_4_ × 7 H_2_O in 200 mL of methanol and acidified with 8 mL of 96% (w/w) sulfuric acid and 8 mL of 85% orthophosphoric acid (w/w). The plate was dried under a stream of cold air and heated at 140 °C for 30 min in a drying chamber. WinCATS software and TLC Scanner 3 (CAMAG, Muttenz, Switzerland) were used in absorption mode at 720 nm for visualization. The lipid spot intensities were integrated (no data smoothing filter was applied) based on the peak area and expressed in arbitrary unit. During method development, iodine vapor was also used as a derivatization agent but only in cases where qualitative information (e.g., separability of the standards) was required.

### Method validation

The validation procedure followed the guidelines of the ICH on analytical validation Q2 (R2) [47]. The criteria used in the validation procedure were specificity, linearity, accuracy, precision, and sensitivity (limit of detection (LOD) and limit of quantitation (LOQ)). This method was validated in the absence of matrices (base calibration).

#### Specificity

Specificity for the identification of the compounds was determined using lipid standards. The HPTLC method was considered specific when the separated compounds had distinct Rf values compared to the neighboring compounds, and no interference was observed between the peaks.

#### Linearity

Lipid standards were used in the range of 100 to 3500 ng band^−1^. After development and visualization, the peak areas were used to establish calibration curves (*n* = 3) based on the least-squares method. The linearity of the method was accepted when the coefficient of determination was ≥ 0.995, when the variable residual plots did not show any trends, and when the *p*-value of the regression was less than the significance level of the *F* statistic ($\alpha$ = 0.05) as it provides sufficient evidence to conclude that the regression model fits the data better than the model with no independent variables.

#### Limit of detection (LOD) and limit of quantification (LOQ)

To determine the LOD and LOQ, the standard error of intercept of the calibration curves (SE) was considered as noise, whereas the signal was evaluated as the regression coefficient (S) and calculated as follows:

$$LOD=\frac{3.3\;SE}S LOQ=\frac{10\;SE}S\;$$

#### Accuracy and precision

The accuracy and precision of the method were assessed using two different concentrations of the lipid standard on different days (*n* = 3) and were therefore considered as inter-day accuracy and precision. To determine the accuracy, 1.100 ng band^−1^, as the midpoint of the calibration curve, was chosen as the target level of 100%. The accuracy was determined at 75% (825 ng band^−1^) and 125% (1.375 ng band^−1^) of the target amount, and the lipid standards were loaded and analyzed using the developed HPTLC method. The amounts recovered experimentally were calculated using linear regression, and the accuracy was expressed as a percentage of the recovered to the theoretical amount:

$$Accuracy [\%]= \frac{recovered\;amount\;[ng\;{band}^{-1}] }{theoretical\;amount\;[ng\;{band}^{-1}]}\bullet 100\%$$

For precision, the lower (150 ng band^−1^) and upper (2050 ng band^−1^) ranges of the calibration curve of each lipid standard were loaded and analyzed using HPTLC. The same target amounts were applied for 3 days in a row for the evaluation of inter-day precision. Precision is expressed as the coefficient of variation of the signal:

$$CV [\%]= \frac{ \sigma AU [-] }{{\overline{x}} AU [-]}\bullet 100\%$$

### Determination of the fatty acid profile

To analyze the fatty acid distribution in the different glycerolipid classes, one microalgae sample per plate (10 × 20 cm) was applied as broad bands of 70 mm and separated as described in “Plate development.” The algae crude extract was spiked with all 13 lipid standards prior to analysis to check the recovery of the bands from the plates based on the obtained fatty acid profiles, which beforehand were determined for all purchased lipid standards. After plate development, the bands were visualized using iodine vapor. The lipid bands were marked with a pencil and scratched with the help of a scalpel and collected in glass tubes with screw-top caps. Three milliliter methanol was pipetted into the tubes and vortexed thoroughly for 1 min to extract lipid molecules from the silica plate. The tubes were then centrifuged at 4.225 × g for 5 min at 4 °C. The supernatant was filtered through a 0.22-µm PTFE membrane and 1 mL of 12 M HCl solution, and a known amount of heptadecanoic acid (C17:0) as an internal standard was added to the reaction tubes, followed by consecutive tempering for 2 h at 95 °C in a water bath. After the fatty acid methyl esterification, 0.4 mL n-hexane (pre-filtered) was added to the tubes along with 3 mL of DI H_2_O (pre-filtered) and vortexed thoroughly for 30 s. After phase separation, the n-hexane phase was transferred to GC vials, and FAME analysis was carried out with the HP 5972 GC–MS device and Hewlett-Packard ChemStation. Ionization was carried out under the voltage of 70 eV at 230 °C and the injector (spitless mode), and the interface temperatures were maintained at 250 °C, and helium was used as carrier gas under constant flow (0.9 mL min^−1^). Gas–liquid chromatography separation was performed on a BPX-70 capillary column (SGE, Melbourne, Australia; 30 m, 0.32 mm inner diameter, 0.25 μm film thickness). The column oven temperature was increased after 2 min of injection from 50C to 179 °C at a rate of 8 °C min^−1^ and kept there for 0.05 min. The temperature then was further increased with 1 °C min^−1^ till it reached 185 °C and held there similarly for 0.05 min. After the initial 8 °C min^−1^ rate was used again till the final, 250 °C, the column oven temperature was reached.

### Data treatment and statistical analysis

The raw data were preprocessed in Microsoft Excel (2019) and imported into the Jupiter Notebook. The processed data were visualized using Pandas, NumPy, SciPy, Matplotlib, and Seaborn packages. In this study, values were interpreted as arithmetic means. Bar charts were used to illustrate the data variations. The heights of the bars represent the arithmetic mean, whereas the error bars represent the standard deviation of repeated measurements. Differences in the lipidomic profiles of the different algal species were assessed using one-way ANOVA (df = 3). Levene’s test was used to assess the equality of variable variances. When the homogeneity test was significant, Brown–Forsythe correction was applied to determine whether a significant difference existed. One-way ANOVA was followed by a post hoc test with Tukey’s correction for multiple comparisons. *P* values lower than 0.05 were considered statistically significant. Statistical analyses were performed using the SciPy software package.

## Results and discussion

Previously published HPTLC/TLC methods for determining the main glycerolipids have indicated limitations. Here, two-dimensional separations were used, which posed a challenging task in terms of densitometric evaluation [27]. In the present study, a one-dimensional method for the selective and sensitive determination of the main microalgal glycerolipid constituents is described and validated in terms of linearity, precision, accuracy recovery, LOD, and LOQ according to well-accepted guidelines [47]. The developed method was also applied to biological samples of four well-known n-3 and n-6 VLC-PUFA-producing microalgae species subjected to glycerolipid analysis.

### Method development

While TLC/HPTLC methods for the separation of carotenoids [48, 49] and neutral lipid fractions [41, 50] are routinely performed with one-dimensional TLC/HPTLC, the separation of the more polar glycerolipids is challenging. Mobile phase systems, mainly composed of chloroform–methanol–water with slight modifications, are widely used to separate phospholipids from animal-derived tissues or human plasma [28, 51]. However, when analyzing plant glycerolipids, it has been observed that in chloroform–methanol–water-based systems, GLs tend to overlap with phospholipids [51]. Therefore, analyzing microalgae is particularly challenging because these cells can be considered as non-specific plant cells without a specialized lipid profile, such as oil seed vs. plant leaf [52]. This overlap of the PL and GL classes presents challenges in both quantitative and qualitative analyses and leaves 2D-TLC state-of-the-art methods in planar chromatography for the separation of microalgal glycerolipids [22, 24, 41].

One-dimensional separation is aimed at accelerating the analysis per plate and allowing the analysis of multiple samples.

**Fig. 2:**
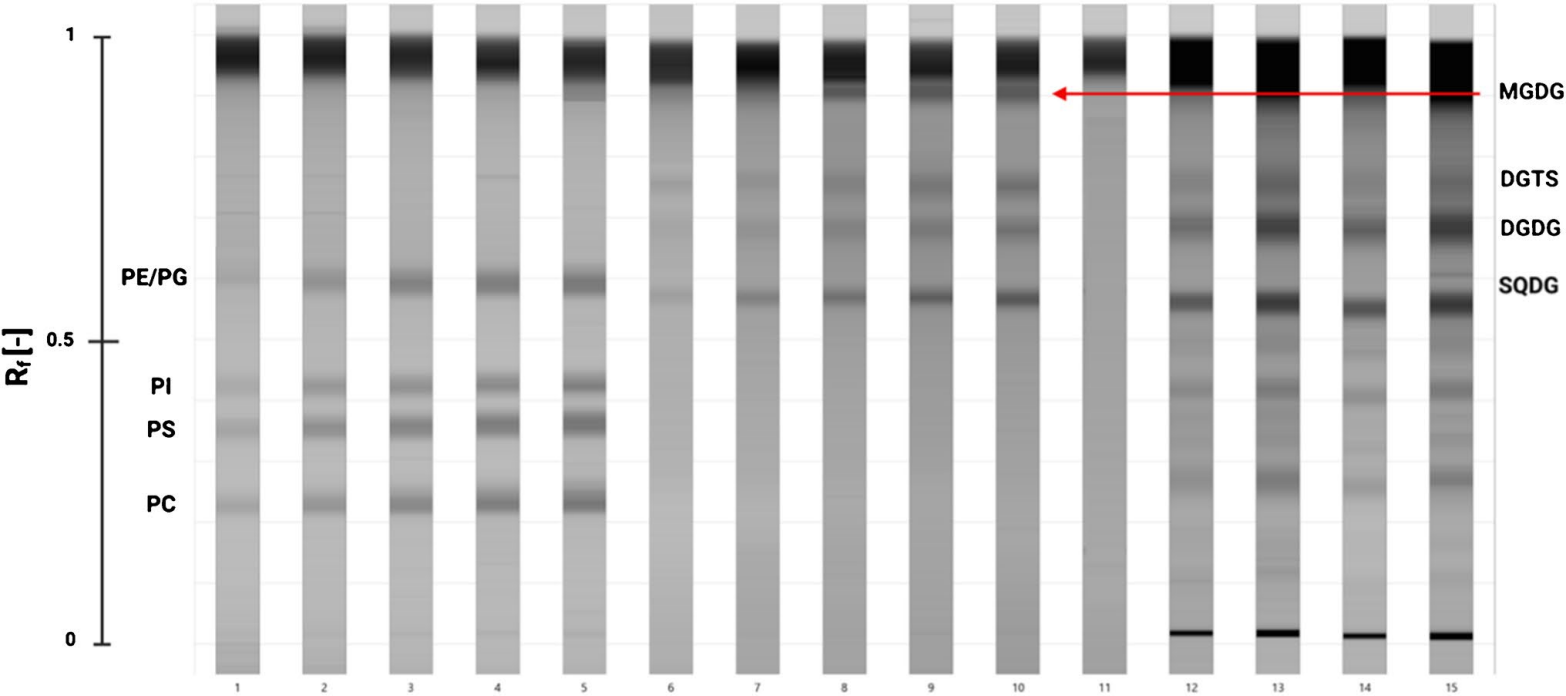
Densitometric scan (top view) from the separation of the PL, GL, and BL lipid class standards using the reported methyl acetate/isopropanol/chloroform/methanol/0.25% aqueous KCl in a 25:25:25:10:9 (v/v/v/v/v) mobile phase system. One to five PL standards in the range of 100 to 2100 ng band^−1^ (the amount of lipid standards between the rows was increased equivalently by 500 ng band^−1^). Six to ten GL and BL standards in the range of 100 to 2100 ng band^−1^; 11, blank; 12–15 crude lipid extracts from *N. granulata*, *P. tricornutum*, *P. purpureum*, and *T. tetrathele*, respectively

Therefore, the first task was to select a mobile phase composition that would allow satisfactory separation of GL, PL, and BL lipid classes. Mobile phase systems consisting mainly of chloroform–methanol–water systems with significant modifications were used in the method development phase. Based on the evaluation of the available data [16, 17, 28, 53, 54], the selected mobile phase systems were chloroform/methanol/acetic acid/water at 28:8:3,2:2 (v/v/v/v), chloroform/methanol/water/ammonia (25%) at 60:34:4:2 (v/v/v/v), and methyl acetate/isopropanol/chloroform/methanol/0.25% aqueous KCl at 25:25:25:10:9 (v/v/v/v/v). First, one lipid standard per band (1.000 ng band^−1^) was applied and developed on 20 × 10 cm plates, as described in “HPTLC.” Based on visual observations, the solvent system consisting of methyl acetate/isopropanol/chloroform/methanol/0.25% aqueous KCl at 25:25:25:10:9 (v/v/v/v/v) showed the greatest potential for the effective separation of the major polar lipid classes of interest. Therefore, a mixture of the five PLs (PC, PS, PI, PE, PG) and three GLs (MGDG, DGDG, SQDG) together with the only available microalgae relevant BL (DGTS) class standard were pooled and used further in the mobile phase method development. Although the applied methyl acetate /isopropanol/chloroform/methanol/0.25% aqueous KCl at 25:25:25:10:9 (v/v/v/v/v) selectively resolved the majority of GLs from PLs and BLs, it was unable to separate PE from PG (Fig. 2). These results indicate that the mobile phase system requires optimization, which allows for the additional separation of the PE and PG lipid classes. Furthermore, the optimized mobile phase system should also increase the separability of PE and PG from the SQDG lipid class, shown in Fig. 2, as no distinct PE/PG and SQDG separation was reached from the applied microalgae extracts.

**Fig. 3:**
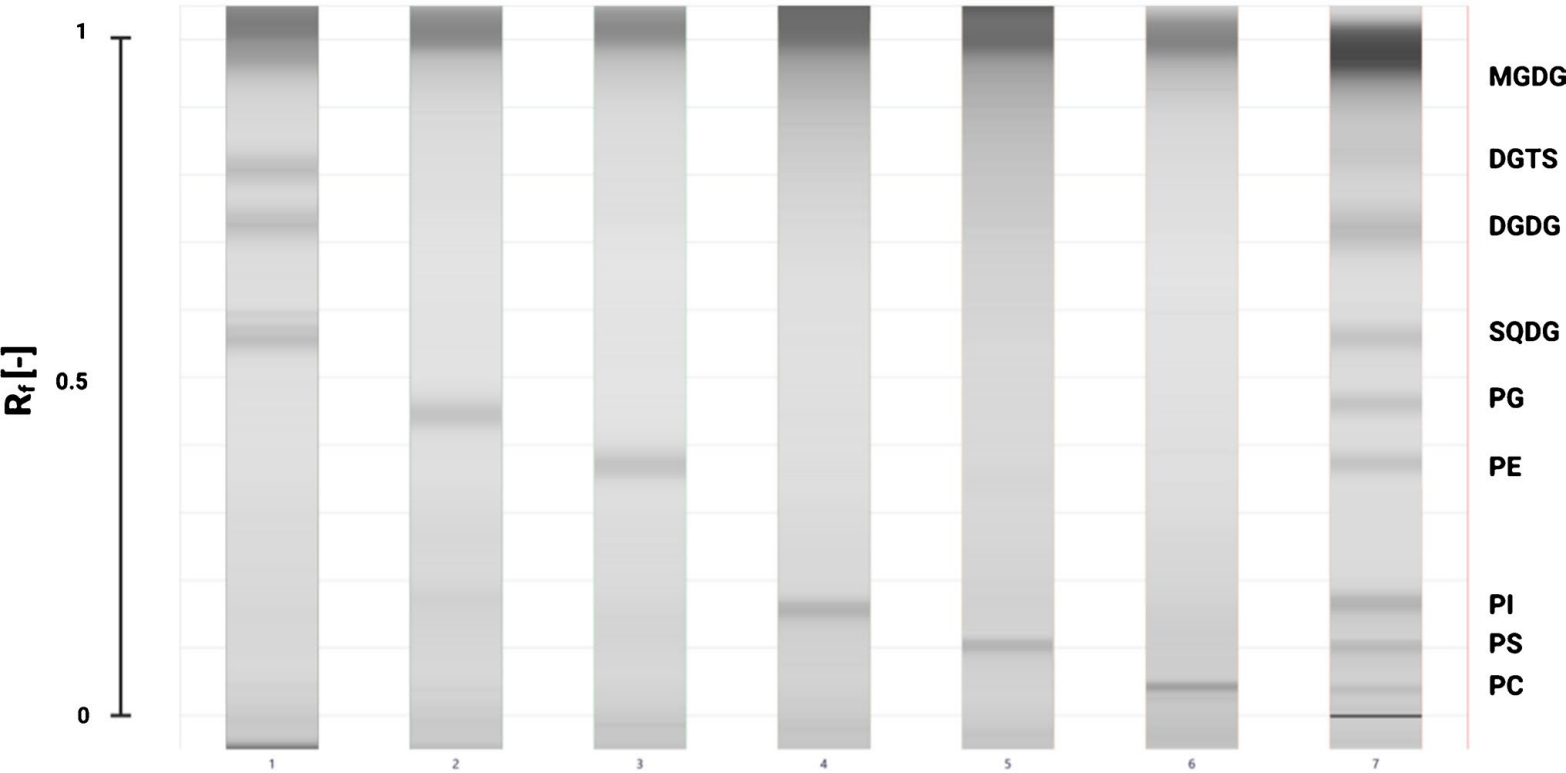
Densitometric scan (top view) from the separation of the lipid standards with the optimized methyl acetate/isopropanol/chloroform/methanol/0.25% aqueous KCl in 25:25:25:10:4.35 (v/v/v/v/v) mobile phase system. 1. GL and BL lipid standards (600 ng band^−1^). From top to bottom: MGDG, DGTS, DGDG, SQDG, 2 PG, 3 PE, 4 PI, 5 PS, 6 PC, 7 crude lipid extract of *N. granulata*

To overcome this problem, the ratio of the most polar solvent of the mobile phase system, 0.25% aqueous KCl, was modified and acidified with glacial acetic acid before applying it in various volumetric ratios. The final ratio, which was proven successful in separating the medium-polar GL, PL, and BLs, was 25:25:25:10:4.35 (v/v/v/v/v) (Fig. 3). The decrease in the ratio of aqueous KCl and acidification lowered the affinity of polar lipid classes to the mobile phase. These modifications also resulted in the separation of PG and PE lipid classes. This was most probably due to the protonation of the amine group of the PE lipid class (Fig. 1), which resulted in a sufficient polarity difference for satisfactory separation from PG. Furthermore, the application of acidified aqueous KCl solution also decreased the peak interference between the SQDG, PE, and PG lipid class. From the crude lipid extracts, three distinguished bands were obtained for SQDG, PE, and PG. Therefore, all separated compounds from the crude lipid extract from microalgae could be assigned to the co-migrating PL, GL, and BL references. ΔR_f_ values no larger than 0.01 were observed between the standards and the separated compounds from the crude lipid extracts (data not shown).

These small differences in the Rf values might arise from the differences in the fatty acid composition of the standards or the presence of oxidized fatty acids, as samples from microalgae are rich in PUFA and highly peroxidation-sensitive. *N. granulata* and *P. purpureum* contain DGTS [55, 56], while in *P. tricornutum*, DGTA is present as BL [24]. However, there is no considerable difference in the R_f_ values of the BL peaks obtained from the microalgae and DGTS standard applied. This suggests that the method presented here cannot separate different BLs; however, it could serve as a useful tool for the estimation of total amount of BLs. Although the polarity of the applied mobile phase system was decreased, the MGDG lipid class eluted almost parallel to the solvent front (see Fig. 2 and Fig. 3), which contained more apolar lipid molecules such as carotenoids, chlorophylls, and neutral lipid fractions of interest (TAG, DAG, MAG, and free fatty acids (FFA)). The co-elution of neutral lipid class molecules confirmed the necessity of using a consecutive mobile phase system. The horizontal broadening of the frontline containing the more apolar molecules was clearly observable with methyl acetate/isopropanol/chloroform/methanol/0.25% aqueous KCl at 25:25:25:10:4.35 (v/v/v/v/v). Thus, frontline zone had to be concentrated again. For this purpose, another mobile phase system containing n-hexane/acetone/isopropanol (80:20:5 v/v/v) was used. The consecutive application successfully separated the less polar fraction from the MGDG lipid class, thus making both the qualitative and quantitative analyses of MGDG lipid class possible.

After the frontline reasonably concentrated to bands again, followed by the drying process, the third mobile phase system consisting of n-hexane/diethyl-ether/glacial acetic acid (70:30:1 v/v/v) was applied on the plates consecutively. After derivatization, 13 distinguished glycerolipid classes were observed (see electronic supplementary material, Fig S1*.)* indicating the satisfactory optimization and applicability of the multiple mobile phase systems for the separation of the main fatty acid containing glycerolipid constituents. To the best of our knowledge, the method presented here is among the first to describe effective one-dimensional separation of 13 major glycerolipid constituents from microalgae [57, 58]. This consecutive solvent development overcomes the limitations of the available HPTLC methods and enables the one-dimensional densitometric quantification of all glycerolipid classes possible.

### Method validation

After the specificity of the method was adjusted and proven using the optimized mobile phase system, lipid references (2.4.1) in the range of 100, 600, 1,100, 1,600, 2,100, 2,600, and 3,100 ng band^−1^ were separated. Using a least-squares linear regression model, calibration curves based on the peak area were established for each lipid standard. A default of linearity, with a secondary polynomial relation, could be observed above 2100 ng band^−1^ in all lipid cases; thus, the upper limit of the linear working range was set to 2100 ng band^−1^. Linear regression models were applied for all lipid classes because no trends were observed in the variable residual plots. The determination coefficients (*R*^2^) of the calibration curves showed a strong correlation coefficient of *R*^2^ > 0.995 (Table 1), and Fisher’s tests for all lipid classes were significant (Fisher, *p* < *0.05*).

**Table 1:** Validation parameters of the defined quantitative 1D-HPTLC-based glycerolipid separation method

No	Lipid class	Adjusted *R*^2^	LOD (ng band^−1^)	LOQ (ng band^−1^)	Accuracy		Precision	
					Applied amount (ng band^−1^)	Recovery (%)	Applied amount (ng band^−1^)	CV (%)
1	PS	0.995	27.32	82.77	825	93.17, …, 108.12	150	3.24
					1375	94.56, …., 105.43	2050	0.99
2	PC	0.995	29.73	90.09	825	95.32, …, 102.67	150	4.94
					1375	94.72, …, 101.32	2050	2.25
3	PI	0.997	21.69	65.74	825	96.21, …, 103.45	150	1.55
					1375	98.34, …, 105.34	2050	1.23
4	PE	0.996	26.26	79.57	825	93.45, …, 106.45	150	6.10
					1375	94.56, …, 107.14	2050	0.55
5	PG	0.998	18.41	55.78	825	92.34, …, 108.39	150	5.98
					1375	95.43, …, 104.84	2050	1.19
6	SQDG	0.996	24.02	72.78	825	96.18, …, 103.45	150	8.54
					1375	98.78, …, 102.98	2050	1.65
7	DGDG	0.997	20.79	62.99	825	93.91, …, 105.69	150	4.58
					1375	95.67, …., 108.77	2050	1.65
8	BL	0.996	25.100	76.07	825	94.39, …, 106.01	150	2.40
					1375	97.56, …, 104.32	2050	1.91
9	MGDG	0.995	27.09	82.08	825	98.59, …, 106.15	150	5.91
					1375	94.21, …, 101.23	2050	2.46
10	MAG	0.997	21.6	65.47	825	96.73, …, 105.61	150	7.26
					1375	97.61, …, 103.49	2050	1.11
11	DAG	0.995	26.54	80.43	825	98.45, …, 105.21	150	6.34
					1375	96.61, …, 104.37	2050	1.47
12	FFA	0.995	27.96	84.72	825	97.54, …, 102.76	150	8.85
					1375	92.32, …, 104.28	2050	1.81
13	TAG	0.995	29.16	71.32	825	91.35, …, 105.21	150	5.94
					1375	96.78, …, 103.89	2050	3.55

The established method also showed high sensitivity, as the LOD values of the separated lipid classes were in the range of 18.41 and 29.73 ng band^−1^, while the LOQ was found between 62.99 and 90.09 ng band^−1^ (Table 1). These findings are superior to those reported by Pinault et al. (2020) in which the lipidome of tumor cells of hepatocyte-isolated mitochondria was explored. The LOD and LOQ limits of similar PL standards obtained here were 10 to 20 times higher, even though the same derivatization protocol was used [27]. Furthermore, as a glycerolipid unspecific derivatization agent was used, we did not encounter such significant deviations in the range of LOD and LOQ. Furthermore, the HPTLC method introduced in this study demonstrates superior performance within lower operational thresholds in comparison to UHPLC-ELSD approaches. Notably, when compared to the method employed by Graeve and Dieter (2009) [59] for quantifying marine-derived PLs and NLs (such as mussels [59] and fishes [30]), the limit of linear working range is reduced by a factor of four. Because the method described by Graeve and Dieter (2009) has been applied to non-photosynthetic organisms, no information regarding the applicability of the method for the separation or quantification of GLs can be concluded. To evaluate the accuracy of this method, 825 ng band^−1^ and 1375 ng band^−1^ lipid class spots were applied and separated. The shift between the theoretical quantity and the amount recovered was evaluated as percentage of recovery, and the results are presented in Table 1. The most extreme values obtained using our method were 93.17 and 108.12% (for PS at 825 ng and 1375 ng, respectively). Recovery rates between 90 and 110% are considered acceptable [33]. In Table 1, the precision is expressed as CV% and is presented for an amount close to the calculated LOQ, 150 ng band^−1^, and close to the limit of linearity, 2050 ng band^−1^. To evaluate inter-day repeatability, lipid standards were applied to the HPTLC plates on different days. Close to the LOQ, the precision was between 1.55 (150 ng PI) and 8.85% (150 ng FFA). The CVs were lower than 3.55% for the amounts used, which is close to the limit of linearity for all PLs (Table 1). As no higher than 10% CV was observed for any lipid class, it implies that the developed method meets the requirement of being precise and accurate in accordance with FDA guidelines in which CV values lower than 15% are accepted.

To the best of our knowledge, this is the first description of an accurate and precise on-plate quantification of multiple PL, GL, BL, and NL classes from microalgae [28, 36]. Therefore, the method could be considered a potential alternative, for example, compared to UPLC-ELSD, for the targeted quantification of glycerolipids, while also offering other advantages such as analysis of multiple samples and lack of pre-chromatographic preparation.

### Case study: glycerolipidome profiling of selected microalgae species

To test the applicability of the method developed, four well-known n-3 and n-6 VLC-PUFA-producing microalgal species of different taxonomic classes were subjected to glycerolipidomics profiling. The GC–MS results (Table 2) confirmed the presence of n-3 and n-6 VLC–PUFAs, among which EPA (C20:5, 7.72 to 40.8 mg g^−1^ biomass) and arachidonic acid (ARA, C20:4, 0.27 to 22.32 mg g^−1^ biomass) were the most abundant PUFAs in all four algal species, whereas DHA (22:6) was only found in *P. tricornutum* (3.70 mg g^−1^ biomass). These values agree with previously published results [60–62] that are commonly derived from total lipid extraction, the hydrolyzation of fatty acids, a methylation, and a subsequent GC analysis. GC–MS-based FA and total overall lipid content determination with chloroform:methanol (2:1 v/v) extraction is a well-established and routinely used tool in microalgal-related research. Despite its importance, the glycerolipid content and the accompanying fatty acid distribution have rarely been published (e.g., *P. tricornutum* [24]*, Nannochloropsis* species [24, 63], *Porphyridium* [64], *Tetraselmis* spp. [65]). Furthermore, lipidomic analysis was primarily carried out at the glycerolipid species level using LC–MS-based quantification methods, which might lead to incorrect quantitative estimations of some lipid species, as has been shown by Johuet et al. [24]. This lack of accurate and quantitative analytical information regarding the glycerolipid positioning of n-3 and n-6 FAs neglects bioavailability assessments and hinders the development of biorefinery value chains for the generation of n-3 and n-6 VLC-PUFA-rich fractions [66].

**Table 2:** Fatty acid composition (mg g^*−*1^ dry biomass) overall lipid content and lipid class-based content of the microalgae investigated in this study. Values are given as the arithmetic mean of the GC–MS, gravimetric, and HPTLC densitometric measurements (*n* = 3). Only fatty acids present in more than 0.01 mg g^*−*1^ dry biomass were considered

Microalgae species	*N. granulata*	*P. tricornutum*	*P. purpureum*	*T. tetrathele*
Class	Eustigmatophyceae	Bacillariophyceae	Rhodophyceae	Chlorodendrophyceae
Lipid content [mg g^−1^]				
Saturated fatty acids				
C12:0	0.42	0.40	0.02	0.47
C14:0	4.52	7.29	0.05	6.87
C16:0	18.11	22.92	22.65	22.33
C18:0	7.60	10.43	2.79	0.92
C22:0	n.d	0.12	n.d	n.d
Monounsaturated fatty acids				
C16:1	18.30	34.42	2.13	23.17
C18:1	6.3	2.07	4.95	0.46
C22:1	n.d	1.67	n.d	n.d
Polyunsaturated fatty acids				
C18:2	2.56	0.49	13.30	2.56
C20:4	7.16	0.27	22.32	2.43
C20:5	40.75	19.1	7.72	33.80
C22:6	n.d	3.70	n.d	n.d
Σ PL content	43.97	42.20	25.00	36.45
Σ GL content	90.24	69.71	70.30	83.34
Σ BL content	13.30	9.62	8.31	11.16
Σ NL content	6.06	18.31	3.68	5.62
Overall lipid content	194.53	200.20	150.45	182.23
Σ Glycerolipid content	153.58	139.84	107.29	136.58
Σ Unidentified	40.95	60.36	43.16	45.65
Unidentified lipids [%]	21.05	30.15	28.68	25.05

**Fig. 4:**
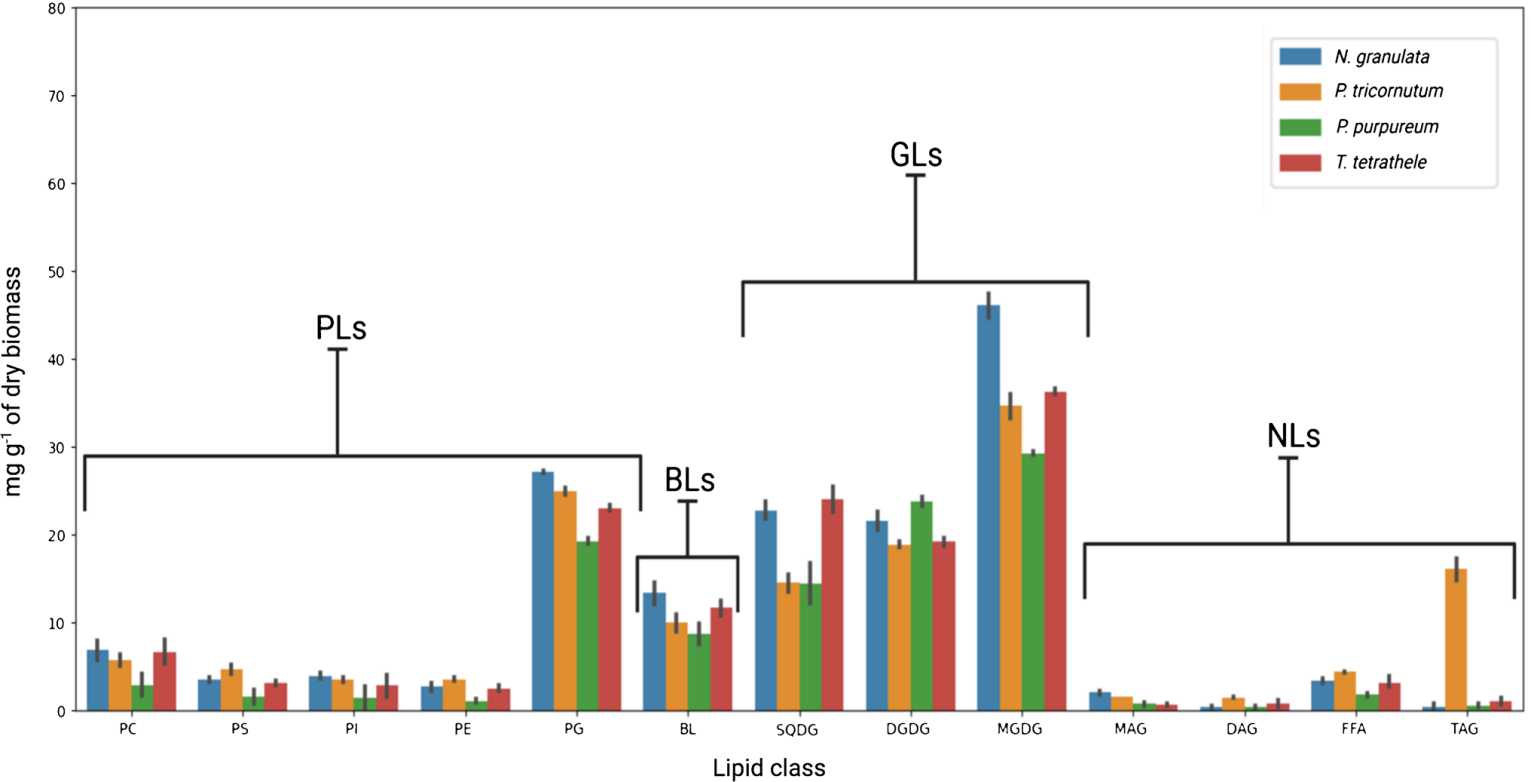
Content of major glycerolipids in the selected microalgae species quantified using 1D-HPTLC. The height of the bar represents the arithmetic mean value of the batches run at the same cultivation temperature, whereas the error bars indicate the standard deviation

Thus, based on the validated method described above, 13 lipid classes were identified in the crude lipid extracts of the selected marine microalgae species. The densitometric measurements revealed that all in all, the photosynthetic membrane-associated GLs were the predominant glycerolipids. However, in the overall GL content, significant differences (*p* < *0.05),* ranging from 69.17 to 90.24 mg g^−1^, were observed among the representatives examined of the four taxonomic classes (Fig. 4, Table 2). Although in microalgae the chloroplast is the most dominant cell organelle, its size and organization differ among the microalgae species owing to the different primary (e.g., *Porphyridium*) and secondary endosymbiotic events (e.g., *Nannochloropsis*) which resulted in a different number of membrane layers within the cells [67]. Therefore, the different levels of surface enhancement organization of the cytoplastic membranes along with differing cell size could serve as a possible explanation for the absolute GL content differences obtained from the studied species [68, 69].

At the glycerolipid class level, MGDG was measured as the most abundant membrane glycerolipid in all four microalgae species, ranging between 33.71 and 46.00 mg g^−1^. This observation is consistent with previous findings; thus, it seems to be a general species-independent agreement that microalgae contain MGDG as the most abundant glycerolipid [70]. However, absolute content differences from previous findings were observed. For instance, Han et al. (2017) showed that MGDG content in another *Nannochloropsis* species, namely, *N. oceanica*, was around 31.9 mg g^−1^, while Johuet et al. (2017) identified MGDG as only 15% in the lipidome of *N. gaditana*. These findings are roughly 30.6 and 36.4% less than the results obtained for *N. granulata* in the present study, respectively*.* These discrepancies in the results may indicate species-specific differences or, more likely, highlight the sensitivity of microalgal metabolic reorientation to different cultivation conditions (e.g., cultivation temperature and light/dark cycles). Therefore, it underscores the importance of monitoring the glycerolipid profile under different cultivation conditions to fully understand the metabolic response of microalgae and to optimize their growth and productivity. Further studies should explore the possible implications of these differences in GL content for the nutritional and industrial applications of microalgae.

PC was the most abundant non-photosynthetic membrane-associated phospholipid, with a content of approximately 0.5-fold that of its structural analog BL (DGTS). PE, PS, and PI were detected as non-photosynthetic membrane-involved glycerolipids, contributing between 1.31 and 3.91% to the overall lipidome (Fig. 4). These results are consistent with the findings of previous studies conducted by Han et. al. (2017) and Johuet et al. (2017). This observation is further supported by the findings of Ferrer-Ledo (2023) and possibly suggests that GL metabolism is more sensitive and species-specific than PL metabolism.

In *N. granulata*, *P. purpureum*, and *T. tetrathele*, TAGs are less than 0.5% of the lipidome, while *P. tricornutum* contained significantly higher (*p* < *0.05)* amount of TAG fractions contributing 7.58% of the lipidome (Fig. 4). This increased level in the TAG fractions might indicate non-optimal cultivation conditions (e.g., temperature, pH) for this specific algae strain, as the formation of TAG under optimal physiological conditions was shown to be minimal, around 2.00% (w/w) of the whole lipidome in the studies of Johuet et al. (2017).

The unidentified lipid classes in microalgae contributed between 21.05 and 30.50% (w/w) to the overall lipidome. Additionally, some non-glycerolipid molecules, which can be extracted using a chloroform:methanol (2:1 v/v) solvent system, were also present. These molecules mainly consisted of photosynthetic pigments (such as chlorophylls and carotenoids), polyphenols, and sterols. The content of these lipophilic molecule species is highly dependent on the cultivation conditions. However, in line with the experimental setup applied in this study, the content of non-fatty acid lipids is reported to be 20 and 45 mg g^−1^ of biomass. Therefore, by summing up the contribution of these reported values and comparing them to the amount of unidentified lipid molecules obtained in this study, it seems that the here-developed HPTLC method is able to comprehensively analyze the main fraction (the glycerolipidome) of the cells whole lipidome.

**Fig. 5:**
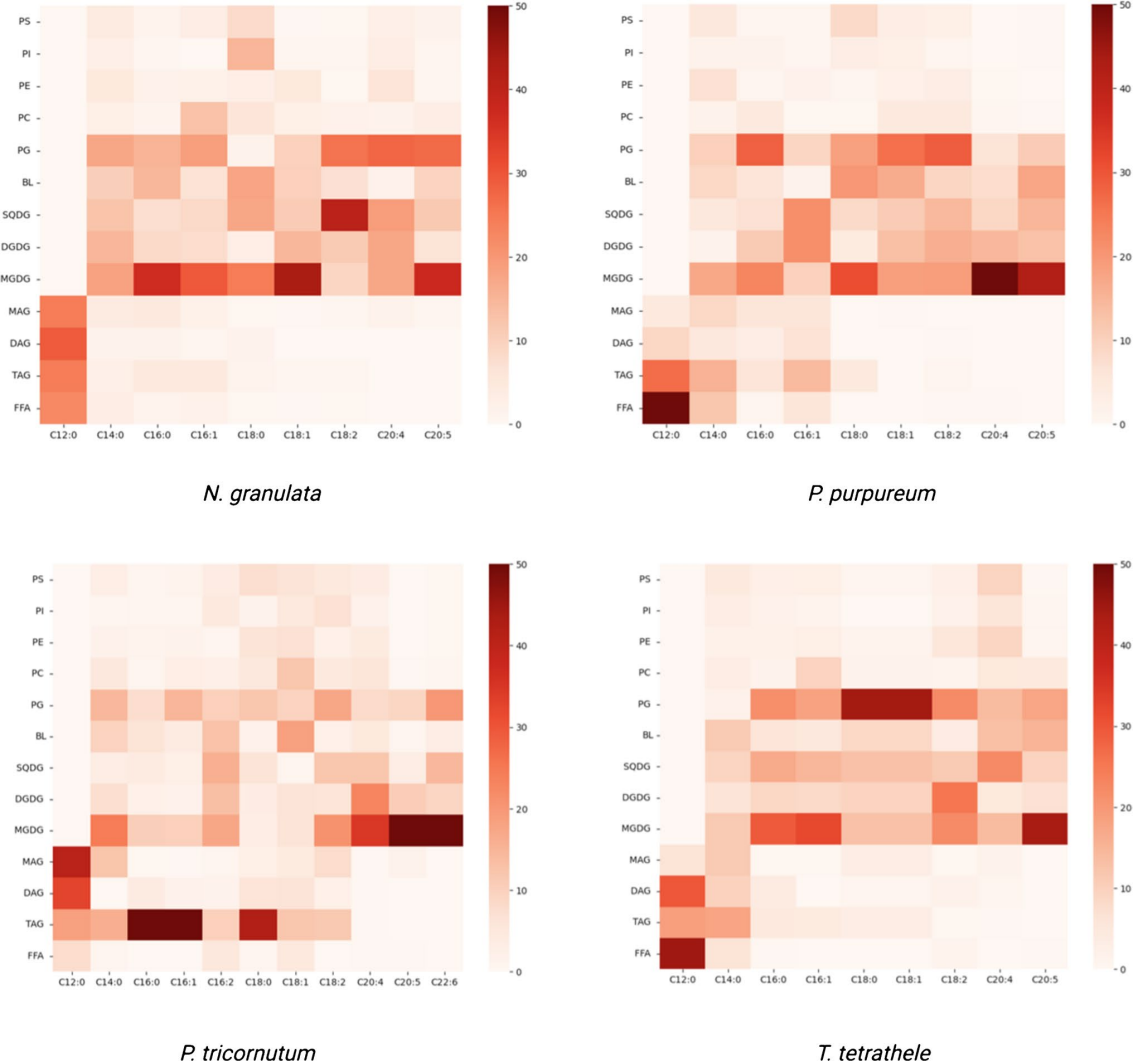
Heatmap of fatty acid acyl chain distribution in glycerolipid classes in selected microalgae species. The values represent mole percentages (mol %). Each row represents the distribution of the given glycerolipid class among the identified FAs of the biomass, while each column represents the mol % contribution of the respective FA in the given glycerolipid class

The HPTLC-GC–MS measurements are summarized in the form of a heat map (Fig. 5), which provides a general overview of the distribution of fatty acids in each glycerolipid class. The results indicate that a variety of fatty acids with different acyl chain lengths and desaturation degrees were unevenly distributed among the glycerolipids. The presence of these fatty acids can have implications for the functionality and properties of lipid classes.

VLC-PUFAs, C20:4, C20:5, and C22:6 (only for *P. tricornutum*) are mainly localized in the thylakoid membrane-associated lipid classes (Fig. 5). In particular, the presence of n-3 and n-6 VLC-PUFAS, independent of the species, was the most abundant in the MGDG lipid class, contributing more than 40 mol% of the fatty acids, which agrees well with previous findings [24, 41, 63]. EPA distribution in the other photosynthetic membrane constituents differed considerably between the species. For instance, the PG lipid class of *N. granulata* was considerably rich in EPA, contributing approximately 27% of the total EPA content, while in the other three examined species, it was approximately 0.5-fold. Despite differences observed in the n-3 and n-6 VLC-PUFAs, the BL glycerolipid group was found to be the most abundant n-3 VLC-PUFA-containing non-thylakoid glycerolipid class, except for *P. tricornutum*. These findings indicate that the most involved glycerolipid classes in n-3 and n-6 VLC-PUFA metabolism were MGDG, DGDG, SQDG, PG, and BL, although taxonomic class-dependent patterns can be observed. The non-photosynthetic PLs contained intermediate chain length fatty acids, except for *T. tetrathele* in which C20:4 n-6 enrichment of these fractions could be observed. Interestingly, the C12:0 fatty acids, independently from the species, were localized in the neutral lipid fractions, mainly in the DAG and MAG fractions. This observation highlights the mediator role of this fatty acid in the elongation and desaturation processes.

Furthermore, the results highlighted the possibility of generating multiple products from the glycerolipids of the studied microalgae, which was previously underestimated. First, the glycerolipidome findings could be used to produce bioactive fractions that are rich in n-3 and n-6 VLC-PUFA. This could be achieved by combining MGDG, DGDG, SQDG, PG, and BL lipid classes, which could fundamentally change the way these fatty acids are currently obtained from fish oil. Additionally, the study suggests that the remaining fractions of the glycerolipids, which are not rich in VLC-PUFA, such as PE, PI, PS, PC, and neutral lipids, could be combined and used as a sustainable alternative to synthetic chemicals (e.g., mineral oils, waxes) in cosmeceutical applications [71] or provide a more ecologically friendly and sustainable way of producing palm oil, which is currently associated with deforestation and other environmental issues [72].

These findings highlight that the distribution of fatty acids is not random, but rather is influenced by the biological processes occurring in each microalgal species. The robust and simple method developed here could serve to gain additional analytical information in comparison to the currently used standard GC–MS method applied both by industry and academia. Hence, a more detailed analysis of the distribution of fatty acids among the different glycerolipid classes and microalgae can lead to a better understanding of lipid metabolism and potential applications in various fields, such as biotechnology, food, and biofuel production. For this purpose, the HPTLC method developed here could serve as a potential analytical tool to further investigate the regulation of these metabolic pathways under varying cultivation conditions.

## Conclusion

We developed and optimized an HPTLC-based method for the quantitative determination of the main glycerolipid constituents (13 in total) in natural extracts, such as microalgae, using a simple on-plate derivatization method. This method is highly efficient, and it does not require pre-chromatographic fractionation of the crude lipid extract, which is routinely performed with solid-phase extraction. Instead, we used consecutively different types of solvent systems to achieve satisfactory separation of the main glycerolipid constituents in a one-dimensional development, enabling the analysis of several samples. Our method provides more comprehensive glycerolipid profiles than the traditional 2D TLC/HPTLC methods, as we were able to quantify a wider range of lipid classes, including phospholipids (PLs), glycolipids (GLs), betaine lipids (BLs), and non-polar lipids (NLs) directly on one plate. The significance of the method developed lies in its ability to differentiate between lipid classes of interest with ease, making it highly applicable in the development of microalgae-based lipophilic high-value products. In addition, we anticipate that our method could pave the way for further research in the field of glycerolipidomics, providing a more efficient way for targeted identification and quantification of a wider range of glycerolipid classes in natural extracts. In the next phase of our research, we will embark on the analysis of individual lipid molecules within each lipid class using the cutting-edge ESI-MSn (electrospray ionization tandem mass spectrometry) technique. This progressive approach will unravel the intricate details of the specific lipid species present in our samples, further enriching our understanding of the glycerolipidome. Therefore, employing this frequently underestimated chromatographic method, an easy differentiation between lipid classes of interest is possible, and its application is conceivable, owing to the modest instrumentation required in the development of microalgae-based lipophilic high-value products.

### Supplementary Information

Below is the link to the electronic supplementary material.

Supplementary file1 (PNG 429 KB)
