# Supplementary figures and images for: Development of a high-performance thin-layer chromatography-based method for targeted glycerolipidome profiling of microalgae
Source: Anal Bioanal Chem. 2024 Jan 4;416(5):1149–64. doi: 10.1007/s00216-023-05101-y (PMC10850188; doi:10.1007/s00216-023-05101-y)

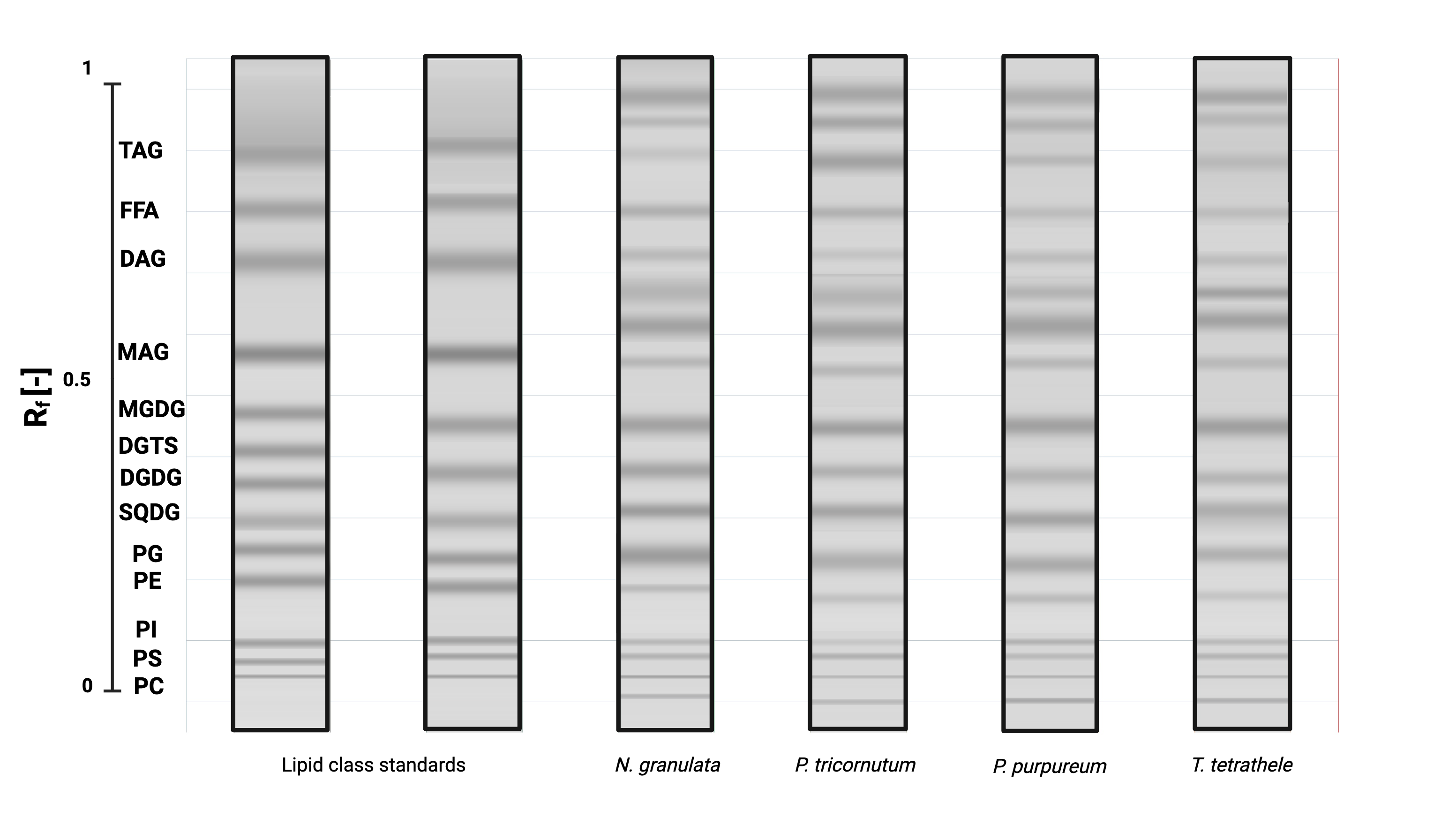

Supplement: Supplementary file 1 — Supplementary file1 (PNG 429 KB) [file 216_2023_5101_MOESM1_ESM.png]
